# G-Protein Dependent Signal Transduction and Ubiquitination in *Dictyostelium*

**DOI:** 10.3390/ijms18102180

**Published:** 2017-10-19

**Authors:** Barbara Pergolizzi, Salvatore Bozzaro, Enrico Bracco

**Affiliations:** 1 Department of Clinical and Biological Sciences, University of Turin, AOUS. Luigi, 10043 Orbassano TO, Italy; barbara.pergolizzi@unito.it (B.P.); salvatore.bozzaro@unito.it (S.B.); 2 Department of Oncology, University of Turin, AOU S. Luigi, 10043 Orbassano TO, Italy

**Keywords:** signal transduction, GPCRs, ubiquitination, cAMP, HERC1, HECT, *Dictyostelium discoideum*, mammals, TORC2

## Abstract

Signal transduction through G-protein-coupled receptors (GPCRs) is central for the regulation of virtually all cellular functions, and it has been widely implicated in human diseases. These receptors activate a common molecular switch that is represented by the heterotrimeric G-protein generating a number of second messengers (cAMP, cGMP, DAG, IP3, Ca^2+^ etc.), leading to a plethora of diverse cellular responses. Spatiotemporal regulation of signals generated by a given GPCR is crucial for proper signalling and is accomplished by a series of biochemical modifications. Over the past few years, it has become evident that many signalling proteins also undergo ubiquitination, a posttranslational modification that typically leads to protein degradation, but also mediates processes such as protein-protein interaction and protein subcellular localization. The social amoeba *Dictyostelium discoideum* has proven to be an excellent model to investigate signal transduction triggered by GPCR activation, as cAMP signalling via GPCR is a major regulator of chemotaxis, cell differentiation, and multicellular morphogenesis. Ubiquitin ligases have been recently involved in these processes. In the present review, we will summarize the most significant pathways activated upon GPCRs stimulation and discuss the role played by ubiquitination in *Dictyostelium* cells.

## 1. Introduction

Signal reception and transduction, a feature that is common and essential to all living organisms, is mainly accomplished by membrane-bound receptors devised to recognize sensory messages from the environment or from other cells.

G-protein coupled receptors (GPCRs) are the largest family of membrane bound receptors and are highly conserved throughout evolution. Features characterizing GPCRs are (a) an extracellular N-terminal, (b) an intracellular C-terminal, and (c) a core domain composed of seven transmembrane regions, hence serpentine or seven transmembrane domain (7TMD) receptors. N-, C-terminal extensions and loops interconnecting the transmembrane regions are critical for signal reception and transduction.

GPCR activation occurs upon ligand binding to their extracellular regions. Differently from receptors with intrinsic enzymatic activity, 7TMD receptors make use of a common molecular switch that is represented by heterotrimeric G-proteins. Ligand binding to GPCR induces conformational changes that eventually lead to membrane recruitment of the G-protein. The activated heterotrimeric G-protein dissociates in the α, β and γ subunits, initiating an intracellular signalling cascade producing different responses. The signalling cascade requires being dynamic and capable of processing, integrating, and coordinating several inputs, all features conceivably made by reversible post-translational modifications, among others phosphorylation, acetylation, methylation, and ubiquitination. In recent years, it has become clear that post-translational modification with the highly conserved 76-residue protein ubiquitin is a process that is even more versatile and complex than phosphorylation, eliciting distinct outcomes within the cell [1]. Indeed, proteomic analyses have suggested that the majority of cellular proteins are at some point modified with ubiquitin [2], which results in either proteasomal degradation, altered function, or subcellular localization. At least three factors contribute to ubiquitin’s ability to act as multi-functional signal: (1) the exquisite specificity of ubiquitin conjugation; (2) the existence of structurally distinct ubiquitin modifications; and (3) the presence of recognition factors that can decipher specific ubiquitin modifications into proper downstream consequences.

**Figure 1:**
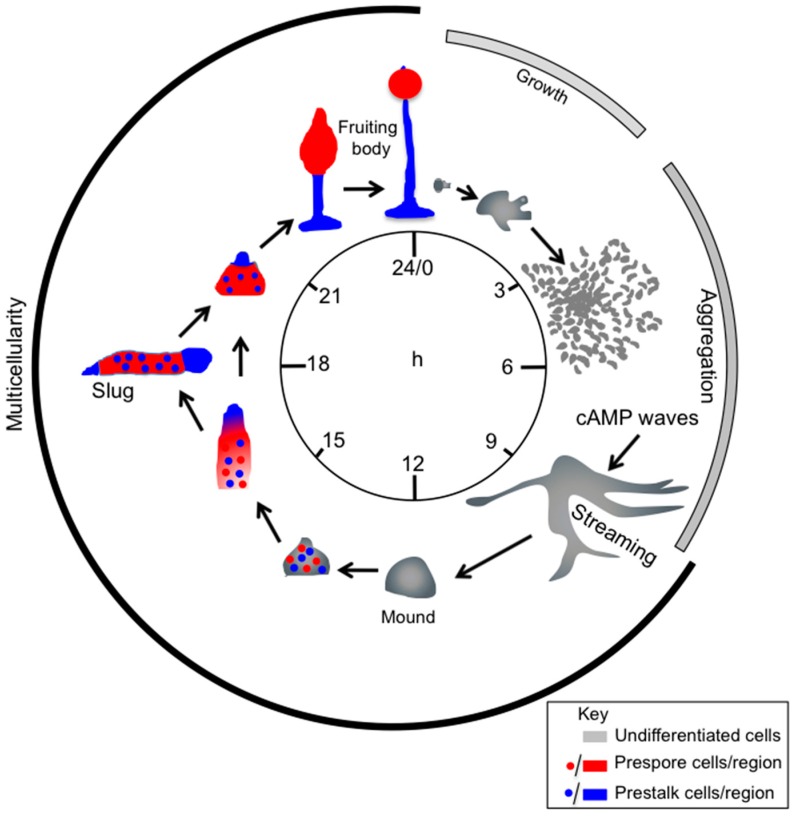
The *Dictyostelium discoideum* life cycle. *Dictyostelium* cells grow by feeding on bacteria and dividing by binary fission. Upon exhaustion of nutrients, starving cells start secreting cAMP, which triggers chemotactic motility and aggregation of cells. Aggregation results in the formation of a multicellular organism, a mound, which forms a tip. The tip is a source of continuous cAMP production and acts as an organizer, regulating morphogenesis and differentiation of cells in at least two cell types, pre-spore (red) and pre-stalk (blue) cells, in a proportion of about 80% and 20%, respectively. Following elongation of the tipped mound and toppling onto the substrate, a sausage-shaped organism is formed—the slug-, which migrates for hours, before culminating into a fruiting body. The slug is coated by a cellulose-like extracellular tissue. During slug migration and fruiting body culmination, pre-spore cells sort out in the front region of the slug and on top of the fruiting body, differentiating into spores within a sorus. The pre-stalk cells undergo vacuolation and death, while forming the stalk of the mature fruiting body. The entire process from starvation to formation of the mature fruiting body takes about 24 h (modified from [11]).

**Figure 2:**
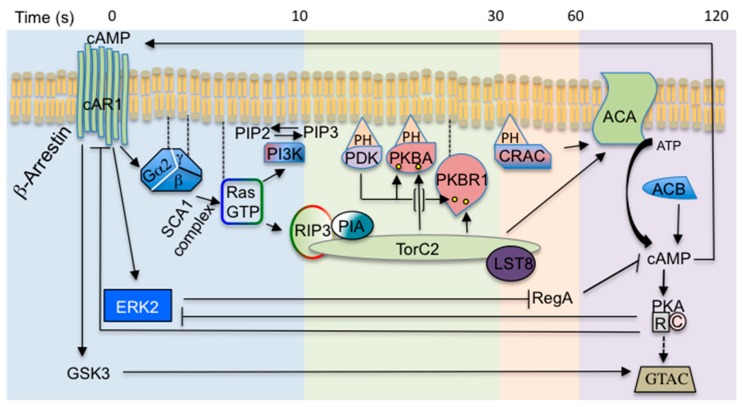
Canonical cAMP pathways during early development and aggregation. Cyclic AMP is secreted by the cells and acts as chemoattractant and hormone-like signal, by binding to the 7-TMD receptor cAR1, thus activating the heterotrimeric Gα2βγ. The G protein then dissociates into Gα2 and Gβγ subunits which activate GTP-Exchange Factors (GEFs) for Ras proteins, leading to activation of PI3K, with localized accumulation of PIP3 and PH domain-containing effectors (PDK, PKBA, CRAC, and PKBR1). RasG activates the TOR Complex 2 -TORC2- via RIP3-Ras interaction. TORC2 in turn mediates phosphorylation and activation of PKBs, which then phosphorylate a number of substrates. PKBs are also phosphorylated by the phosphoinositide-dependent protein kinase (PDK). PKBs phosphorylation sites are denoted by yellow dots. The Rictor homolog Pia and CRAC synergistically activate the adenylyl cyclase ACA that converts ATP into cAMP, thus leading to PKA activation, GtaC phosphorylation and finally expression of genes required for aggregation. Most of the cAMP produced is secreted extracellularly, giving rise to cAMP relay. Cyclic AMP binding to cAR1 also controls activation of the Extracellular signal-Regulated Kinase-2, ERK2, that is in turn inactivated by PKA. ERK2 then phosphorylates the cAMP-specific intracellular phosphodiesterase RegA. The activation of the cAR1 also induces translocation of cytosolic β-arrestins to the plasma membrane to form a complex with cAR1. These stimulatory and inhibitory pathways are responsible for the oscillatory production of cAMP pulses, which are essential for regulation of gene expression. Solid arrows and T-bars represent positive and inhibitory loops, respectively. Dashed arrow denotes putative target activation. The timing of cAMP signalling responses, following a cAMP pulse, is indicated in seconds (top). For further details see text. Abbreviations: PI3K, Phosphatidylinositol-3 Phosphate Kinase; PIP3, Phosphatidylinositol-3 Phosphate; PH, Pleckstrin Homology; PDK, 3-Phosphoinositide-Dependent protein Kinase; CRAC, Cytosolic Regulator of Adenylyl Cyclase; PKBA, Protein-Kinase-B A; PKBR1, Protein-Kinase-B Related 1; PKBs, Protein-Kinase-B members; PKA, Protein-Kinase-A; GtaC, GATA transcription factor family member C.

In GPCR, signalling leading to chemotaxis much of our current understanding come from studies with the social amoeba *Dictyostelium discoideum* and mammalian neutrophils [3]. *Dictyostelium* cells grow as single cells feeding on bacteria. To face food depletion *Dictyostelium* employs an elegant survival mechanism. The solitary vegetative cells stop growing and set a tight developmental schedule (Figure 1). Initially cells undergo chemotactic aggregation driven by secreted cAMP, eventually giving rise to multicellular aggregates. This process is the result of intricate cross-talk pathways as depicted in Figure 2. The ability to synthesize the proteins necessary for the production, detection and degradation of cAMP is acquired gradually. After a few hours of starvation, cells start releasing periodically cAMP, giving rise to cAMP oscillatory waves that propagate over the substratum, attracting neighbour cells, which move towards the cAMP source and, in turn, release themselves cAMP. The relayed signal allows for recruiting the more distal cells, creating inward waves of migration. Concomitantly with the ability to respond to cAMP, the cells become more adhesive, attaching end-to-end in streams, and giving rise to mound-like tight aggregates. The mounds develop a tip and start elongating, toppling down on the substrate, forming slug-shaped organisms capable of migrating, guided either by light (phototaxis) or by temperature (thermotaxis). During mound and slug formation, cell differentiation in at least two distinct populations, pre-spore and pre-stalk cells, takes place. In the case of prolonged food deprivation, the slugs undergo culmination, forming a mature fruiting body consisting of a spore mass hold up by a slender tapering stalk made of highly vacuolated, dead cells. The mature fruiting bodies usually measure between 1.5 and 3 mm in height. The complete developmental process takes place in about 24 h [4]. When food sources become available again, spores germinate and start proliferating once more as solitary amoebae.

Because of its haploid genome, a real boost with *Dictyostelium* research was offered by its easy genetic manipulation, i.e., gene inactivation/silencing by homologous recombination, Reference [5], and later on by random plasmid insertional mutagenesis (REMI: Restriction Enzyme Mediated Integration) [6]. The complete sequencing and analysis of the 34 Mb *Dictyostelium discoideum* genome [7] revealed that many proteins are highly conserved, often showing higher homology to mammals, including humans, than to fungi or plants. The haploid nature of the genome is useful in that phenotypes are immediately obvious after mutagenesis. In addition, because of the mutual exclusivity of cell division and development, mutations that block development have no effect on cell growth, and are therefore essentially conditional. For all of these reasons, *Dictyostelium* has become a preferred model for studying cell signalling, chemotaxis, motility, cell-cell adhesion, phagocytosis, host-pathogen interactions, and as a model for diseases [8,9,10].

## 2. The *Dictyostelium* Ubiquitin Landscape: A Short Glance

The tagging of a substrate with ubiquitin is finely tuned and occurs through the sequential actions of three enzymes [12]. First, ubiquitin is activated by an ubiquitin activating enzyme (E1) in an ATP-dependent way. Then the activated ubiquitin is transferred to the active site cysteine (Cys) residue of an ubiquitin-conjugating enzyme (E2), and then ubiquitin is generally transferred from the E2 to the N-amino group of a substrate lysine (Lys) in an E3-dependent manner. The conjugation cascade is hierarchically organized, usually consisting of a single E1, a limited number of E2s and many E3s, playing a key role in target protein selection through its ability to recognize a structural motif presented by its [13] cognate substrates [14,15]. Because of this, while E2 are characterized by a highly conserved domain, E3 are a much more diverse group of proteins in terms of size and domain structure [16]. According to their domain composition and catalytic activity, the E3 class ubiquitin ligase can be classified into three major families: HECT (Homologous to the E6AP Carboxyl Terminus), RING finger, and RBR (Ring-Between-Ring). The RING finger family represents the largest group of E3 with ~600 members in mammalian genomic databases. They do not have a direct catalytic role in protein ubiquitination, but act as scaffolding partners facilitating the interaction between an E2 and the different substrates. Unlike the RING finger E3, mammalian HECT E3s family is smaller, accounting for ~30 different members characterized by a unique catalytic domain (HECT domain) functioning in a similar manner to the E1 and E2, since a central Cys residue acts as acceptor for ubiquitin, which is in turn transferred to the substrate. RBR family accounts for ~15 members in mammals working similarly to the HECT E3 ubiquitin ligases. Besides their ligase activity towards different protein substrates, E3 ligases may also catalyze the formation of free poly-ubiquitin chains [17], whose precise role still remains poorly characterized. The ubiquitination is a reversible process regulated by numerous deubiquitination enzymes (DUBs), which act as proteolytic enzymes by cleaving the covalent bond formed either between the ubiquitin and the substrate or between ubiquitin molecules within a polyubiquitin chain [18].

The *Dictyostelium* genome harbours several ubiquitin and ubiquitin regulators (ubiquitin ligases and de-ubiquitynilating enzymes) encoding genes. *Dictyostelium* genome mining reveals that the ubiquitin genes constitute a multigene family of eight members, encoding tandem repeats of 76-aminoacid ubiquitin unit. In addition, there are four more genes containing a single ubiquitin repeat two of which are fused at its 3′ end to an unrelated extension rich in basic aminoacid residues with a remarkable similarity in the sequences to that of yeast and mammalian counterpart [19]. The expression of the *Dictyostelium* ubiquitin-encoding genes is developmentally regulated and sensitive to thermal shock, heavy metal exposure, and protein synthesis inhibition [20].

In an effort to make a sort of inventory of the genes encoding for the ubiquitin regulators, we identified approximately 50 putative members of the DUBs (Table S1). Protein deubiquitination by DUBs can either antagonize or facilitate substrate presentation to the proteasome. In the past, the *Dictyostelium* 20S proteasome complexes have been isolated, and their proteolytic activities characterized [21]. The 20S complexes have been found both in the cytosol and, in higher concentrations, in the nucleus. More recently, a component of the 19S regulatory complex has also been characterized, displaying a range of proteasome activation broader than that of the metazoan [22]. Other than the proteasome complex, *Dictyostelium* DUB family members did not receive much attention in the past, though they appear to be crucial in regulating development but not growth. Lindsey et al. [23] identified a deubiquitinating enzyme, ubpA, that specifically disassemble polyubiquitin chains, establishing that developmental transitions require the degradation of specific proteins, and that this process in turns needs free ubiquitin monomers resulting from the disassembly of freely cytosolic polyubiquitin chains. A second DUB member, ubpB, has been described in Dictyostelium. Though, *ubpB*, mRNA expression is developmentally regulated showing higher levels during the aggregation and the mound stage, its role appears to be restricted to the late post-aggregative phases where it is required for the proper pre-spore cell patterning. Interestingly, ubpB does physically interact with the Fbox and WD40 domains of the Mitogen-activated Protein Kinase Kinase Kinase alpha MEKKα, thus controlling its stability. From the experimental evidence provided it is very likely that ubpB acts as a deubiquitinating enzyme for anchored polyubiquitin chains [24].

According to our inventory, in the *Dictyostelium* genome, we identified several ubiquitination machinery components belonging to the three ubiquitin ligase families: E1, E2, and E3.

**Figure 3:**
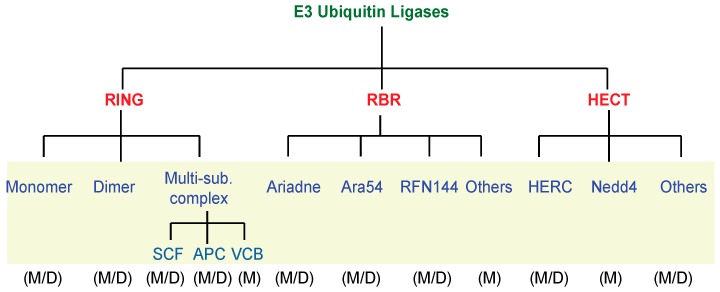
Summary of the E3 ubiquitin ligases family members. Mammals (M) and *Dictyostelium* (D) E3 ubiquitin ligases members have been classified and compared according to their structural features.

Close to ten E1 members can be found in the *Dictyostelium* genome, and to date none of them has been characterized (Table S1). The E2 family accounts for approximately 30 different members (Table S1). Currently, only one of these, UbcB, has been characterized. *UbcB*-null cells are blocked at mound stage and never form fruiting bodies indicating a role for this specific E2 member in controlling the developmental transition at late post-aggregative stage [25]. The E3 family is the most numerous, with around 140 different members split into three sub-families, RING, HECT, and RBR, according to their structural features (Figure 3, and Table S1). As depicted in Figure 3, almost all members of the mammalian sub-families are represented. As it occurs in mammals, the *Dictyostelium* RING sub-family possesses either monomer, dimer or multi-subunit members, but differently from mammals members of the heterodimer and of the VCB-Cul2 (Von-Hippel-Cul2/elongin) multi-subunit complex are missing.

Few *Dictyostelium* E3 family members have been characterized, mostly belonging to the RING family, either acting as monomer (e.g., Cbl-orthologue and MEK Interacting Protein-MIP) [26,27], or components of the multisubunit RING E3, such as cullin members [28] and a F-box WD40 containing protein (FBXW) [29]. Less characterized are the RBR and HECT family members. We have identified 10 genes encoding putative RBR E3 ubiquitin ligases, including the orthologues of ARA54 and RNF144 subgroups, but by now only a single member, belonging to the Ariadne subgroup [30], has been characterized. Among the E3 ubiquitin ligase sub-family, that of HECT is the most heterogeneous in terms of size ranging from 70–80 to more than 500 kDa. We identified five members of this family. Structural analysis reveals that most of them cannot be ascribed to the conventional mammalian counterparts because apart the HECT domain, the other domains are not structurally conserved. Currently, two members of this subfamily, namely HfnA [31] and HectPH1 [32], have been characterized. While the first is structurally related to the mammalian counterpart HECT-filamin, consisting of a filamin domain located at the N-terminus and a HECT at its C-terminus, HectPH1 can be considered a non-conventional member of the so-called large HERCs because of its very large size of 588 kDa. Mammalian large HERCs are defined because of their single HECT domain, at least one SPRY domain and a pair of the Regulator of Chromosome Condensation (RCC1)-like domains (RLD). RLD is a structurally conserved, yet functionally very versatile domain, whose roles may include interactions with other proteins or phospholipids [13]. Interestingly, *Dictyostelium* HectPH1 shares with the large HERCs all the structural features but it lacks the RLD, which is replaced by a PH domain that might act functionally in a similar manner to the RLD. Indeed, it has been shown that the isolated PH domain fused to GFP is enriched in the nuclear membrane and the nucleus, though some labelling in the plasma membrane has been observed. Structurally, the PH domain is located close to the HECT domain, and the latter is homologous to the HECT domain of the mammalian HERC1 (HECT and RLD domain-Containing E3-ubiquitin ligase protein 1) [32].

## 3. G-Protein Coupled Receptors and Their Molecular Switches

The *Dictyostelium* genome harbours 55 GPCR encoding genes [33] that belong to four of the six GPCR families (Figure S1). The figure is enormous if compared to the only 3 GPCRs of the yeast *S. cerevisiae*.

To date, with the exception of very few GPCRs [34,35,36,37] only the cAMP binding receptors (cARs) have been addressed extensively [38]. This family encompasses 4 well characterized (cAR1-4) and 3 less well characterized cAR-like members [39]. cARs bind cAMP with different affinities, being cAR1 and cAR3 those with higher affinity, and transduce the signal throughout different G-proteins. As classical GPCRs, they share the trademark of seven transmembrane spanning domains and differ mainly in their tails. *Dictyostelium* cells express cAR1 at the very early developmental stage. The receptor exhibits a high affinity for cAMP, enabling cells to sense very low levels of secreted cAMP. Its occupancy has three main consequences: (1) chemotaxis of cell towards the cAMP source; (2) changes in gene expression; and (3) synthesis and secretion of more cAMP. Upon ligand binding the receptor undergoes conformational changes, exposing cytoplasmic sequences that interact with heterotrimeric G proteins. In *Dictyostelium* there are 12 genes encoding Gα subunits but only single genes encoding for Gβ and Gγ [40,41,42]. The inactivation of the single *Dictyostelium* Gβ gene has been paramount for identifying GPCR-dependent, but G protein-independent, signalling pathways [41]. cAR1 also regulates intracellular effectors in a G protein independent manner (i.e., Ca^2+^ uptake, GSK3, and ERK2 activation). The heterotrimeric G protein mediating cAR1 signaling is Gα2βγ, and its activity is antagonized by the Gα9 subunit that functions attenuating the multiple pathways coupled to chemoattractant receptor [43]. Most of cellular responses triggered by cAMP, following its binding to cAR1, are transient. This means that receptors and the signal transduction machinery must carry out an exceptionally efficient process of de- and re-sensitization. As it happens in animal cells, at the very early phases of signal transduction cAR1 becomes phosphorylated on several serine (Ser) residues [44,45]. Phosphorylated cAR1 has reduced cAMP affinity [46], but its precise meaning in the adaptation process and the related kinase involved in such modification have been matter of debate over the past 2–3 decades. Recent observations [47] provide experimental evidence indicating that at least some adaptive processes depends on receptor phosphorylation.

Activated cAR1 acts as a docking site for arrestins, like as it happens in animal cells. *Dictyostelium* possess at least six putative arrestin family members. Three of them have been characterized: AdcA plays a role in the formation of early endosomes [48], while AdcB and AdcC regulate the frequency of cAMP oscillations, and may link cAR1 signalling to oscillatory ERK2 activity. One of the family member, AdcC, physically associates with ERK2 [49]. A few seconds upon ligand stimulation, AdcC is transiently and rapidly recruited to the inner face of plasma membrane, where it associates with the cytoplasmic tail of cAR1 in a Gβγ independent way but, conversely to mammalian cells, the event is phosphorylation independent. An *adcB*/*adcC*-null strain displays impaired cAR1 internalization, indicating that arrestin is required for receptor internalization. Arrestin null mutants, however, do not display obvious chemotactic defects. For quite long time, arrestins have been linked to receptor down-modulation, as being actively involved in receptor mediated endocytosis. It is now well recognized that receptor internalization does not necessarily correspond to desensitization. Indeed, GPCR desensitization occurs much faster than internalization, indicating that it depends on its phosphorylation status and only later on arrestin. In line with this, it has been observed that GPCRs continue signalling even at the level of the endosomal compartment [50,51]. Besides phosphorylation, it has recently emerged that ubiquitination is important for regulation of GPCR signalling. Indeed, ubiquitination of either the receptor or arrestin is necessary for receptor degradation and internalization, respectively [52,53,54]. Agonist-dependent GPCR ubiquitination can occur either via direct binding of the ubiquitin E3 ligase to the receptor (e.g., phosphorylated C-X-C chemokine Receptor type 4 -CXCR4- binds E3 ubiquitin ligase AIP4) or indirectly through arrestin, which is acting as an adaptor bring the E3 ubiquitin ligase in close proximity of the receptor (e.g., β2Adrenergic-Nedd4 interaction). A similar mechanism might take place for cAR1 in *Dictyostelium*. Indeed, AdcC has been found to physically interact with two E3 protein ligases, one of which belonging to the HECT family. Furthermore, the relevance of ubiquitination at GPCR level is highlighted by the fact that *Dictyostelium* cAR3, a cAMP receptor expressed in the late aggregation stage, interacts with the CSN5/Jab subunit of COP9/signalosome complex [55]. COP9/signalosome functions as a large multiprotein complex, regulating the ubiquitination process and, together with the accessory 19S, may assist the entry of the substrate into the proteasome 20S complex. The inactivation of the *Dictyostelium* genes encoding for the CSN2 and CSN5 COP9 subunits display lethal phenotype indicating that COP9/signalosome complex is required for cell growth.

Currently, the knowledge on G-protein regulation by co- and post-translational modifications is still scant. However, it is well known that both the Gα and Gγ subunits are subjected to irreversible lipid modifications, necessary for their docking to the plasma membrane, and to transient phosphorylation linked to regulation of the heterotrimer integrity [56]. In addition to these modifications, it is lately emerging that mammalian G–protein stability is controlled by ubiquitination [57,58].

## 4. Navigating Downstream of G-Protein: Ras, PI3K and TORC2

One of the earliest responses downstream of activated G-protein in *Dictyostelium* is the stimulation of the Ras proteins. Indeed, activation of RasG and RasC, orthologues of mammalian K- and H-Ras, at the leading edge of cAMP-stimulated polarized cells is the earliest measurable signalling event, occurring within seconds. RasGEF is rapidly activated, leading to an initial surge in membrane associated RasGTP. The response then adapts within 30 s and the level of membrane associated RasGTP returns to its initial state, as the activity of RasGAP increases and suppress that of RasGEF. The concurrent activation of both RasGEF and RasGAP confers to the network ultrasensitivity [59]. Ras activation is immediately followed by the RasGTP-dependent activation of the phosphatydylinositol-3 phosphate kinase (PI3K), which converts phosphatidylinositol phosphate PIP2 to PIP3. PIP3 represents a docking site for transient recruitment of the PH domain-containing proteins CRAC (Cytosolic regulator of Adenylyl Cyclase), PDK (3-Phosphoinositide dependent Kinase), and PKB (protein kinase B). *Dictyostelium* possesses two PKB/Akt, PKBA and PKBR1. Like animal Akt, PKBA harbours a PH domain, whereas PKBR1 is membrane anchored. The latter one appears to play a major role in signal transduction than PKBA. The PIP3 levels are finely controlled by the lipid phosphatase PTEN [60]. Differently from animal cells, where at the cytosolic surface of plasma membrane, PDK and TORC2 get in close proximity to PKB by means of transient PIP3 platforms, thus allowing the phosphorylation of crucial PKB Ser residues, in *Dictyostelium* the TORC2 activation is PIP3 independent, as PKBR1 is membrane anchored. A contribution to TORC2 targeting to the inner side of plasma membrane derives from Ras proteins. RasC, RasG, and Rap1 have been shown to interact with TORC2. The first one via the TOR kinase domain, RasG, and Rap1 indirectly throughout the Ras binding domain of the *Dictyostelium* Sin1 orthologue, RIP3 [61,62]. Phosphorylated PKBs are thus activated, and in turn phosphorylate different intracellular substrates including talin-B, RasGEFs, PI5K, eventually reorganizing the actin cytoskeleton during chemotactic motility. Selective inactivation of the different TORC2 components, namely Pia/Rictor and lst8, also led to dramatic reduction in Adenylyl Cyclase A (ACA) activation [61], indicating that a preformed TORC2 is necessary to regulate ACA activation, and thus cAMP relay. These data show that TORC2 acts as integrating platform of multiple distinct signalling pathways to control cell aggregation and the formation of a multicellular organism.

Effectors acting downstream of Ras include the MEK-kinase, MEK, and ERK members. The *Dictyostelium* MEK1 (DdMEK1), activated via cAR1 upon cAMP binding, is required for proper aggregation. Indeed, *DdMEK1* null strain produces extremely small aggregates giving rise to significantly smaller fruiting bodies. Small aggregates are a consequence of impaired chemotaxis due to inability to sense cAMP, though ACA, but not guanylyl cyclase, activity was rather normal compared to that of wild-type [63]. DdMEK1 is rapidly and transiently SUMOylated in response to cAMP stimulation. Such modification is required for translocation from the nucleus to the cellular cortex at the leading edge of the polarized chemotactic cell, and it coincides with the active form of DdMEK1. By contrast, its nuclear retention is controlled by ubiquitination. A MEK Interacting Protein (MIP), which belongs to the E3 ubiquitin ligase family, acts at nuclear level in a proteasome-independent fashion by ubiquitinating and thus sequestering DdMEK1 within the nucleus [27]. Additionally, the stability of the *Dictyostelium* MEK Kinase (MEKKα), a developmentally regulated MAP Kinase family member that harbours F-box and WD40 repeats, and controls timing and spatial developmental patterning, is regulated by ubiquitin-mediated degradation [24].

Within this context, components other than MEK1 and MEKKα might be under tight control of ubiquitination. In mammalian cells, the activities of several of these molecular players are regulated by ubiquitinationIndeed the PHD domain of the mammalian MEKK1 a close orthologue of the Dictyostelium MEKKα, that exhibits a RING finger-like structure, displays E3 ubiquitin ligase activity toward ERK2 in vitro e in vivo. Moreover, both MEKK1 kinase activity and docking motif on ERK1/2 were involved in ERK1/2 ubiquitination. MEKK1 functions not only as an upstream activator of ERK and JNK through its kinase domain, but also as an E3 ligase through its PHD domain, providing a negative regulatory mechanism for decreasing ERK1/2 activity [64]. It is not known whether in *Dictyostelium* ERK2 regulation occurs similarly, and so far, no experimental data support this hypothesis. However, due to the structural similarity between H.s. MEKK1 and D.d. MEKKα a similar mechanism might be envisaged in *Dictyostelium* as well.

Additionally, Ras family members can be sorted-out to distinct membrane compartments by means of differential ubiquitination [65].

Similarly, the Akt activation includes several major steps tightly regulated by kinases, phosphatases, and E3 ubiquitin ligases. Actually, in mammals the phosphorylation of the PKB Ser473 is catalyzed by kinases other than TORC2, such as the DNA-Polymerase Kinase DNA-PK [66]. The action of the PKB activating kinases (PDK, TORC2, and DNA-PK) is counteracted by phosphatases, including PP2A and PHLPP [67]. Whereas, the first one appears to be responsible for the dephosphorylation at the level of the activation loop (Thr308 in mammals) the second is hydrophobic motif specific (Ser473 in mammals) and, notably, its activity is under the control of an E3 RING ubiquitin ligase [68]. All of these events occur at the plasma membrane level, where Akt is recruited by means of its PH domain. Crucial to promote PKB/Akt plasma membrane recruitment, and its subsequent activation is ubiquitination, which enables the binding of the PH domain to PIP3 [69]. In addition to its role in activating Akt, ubiquitination can attenuate Akt signalling leading to proteasome degradation of the kinase [67]. Rictor itself is modified by either phosphorylation or ubiquitination. Indeed, proteasome chemical inhibition increases Rictor ubiquitination and its levels. Rictor degradation is mediated by FBXW7-ubiquitination and GSK3. High levels of Rictor induce increase of the TORC2 activity, and in turn dysregulated Akt activity [70]. Additionally, Rictor has also been shown to associate with other proteins independently from mTORC2, such as cullin-1 and F-Box, and WD repeat domain, containing 7 (FBXW7) members of the E3 RING multi-subunit ubiquitin ligase complex, implying that Rictor could mediate other biological functions outside of mTORC2 [71]. In *Dictyostelium,* nothing similar has been reported yet, although several of the molecular players are conserved and shared with mammalian cells. A potential role of HectPH1 in these processes will be discussed further below.

## 5. Intracellular cAMP Signalling Converges to PKA

*Dictyostelium* cellular aggregation requires the synchronous secretion of cAMP in an oscillatory fashion [72]. Surrounding cells respond both by moving chemotactically and by secreting more cAMP, thus relaying the signal to cells further away. The cAMP oscillations are necessary for regulating the cellular and biochemical changes, including gene expression, required by cells to develop properly.

In *Dictyostelium,* three genes (*acaA*, *acrA,* and *acaG*) encoding distinct adenylyl cyclases have been characterized and shown to be expressed at different stages of development, with *acaA* and *acrA* encoded proteins being involved in aggregation and postaggregative development [73,74]. *acaA* encodes a 12 transmembrane protein (ACA) accumulating shortly after the initiation of development and functioning in relay of extracellular cAMP pulses. Its enzymatic activity is positively regulated by TORC2. *acrA* encodes a protein, ACB, with domains related to histidine kinase response regulators, and a C-terminal portion containing the adenylyl cyclase catalytic domain [74]. ACB is present at low levels in vegetative cells, and starts accumulating during the aggregation and remains high throughout postaggregative development [74,75].

A small fraction of the cAMP produced by ACA, and presumably by ACB, remains within the cell to activate the cAMP-dependent protein kinase A (PKA). PKA controls a number of diverse regulatory pathways. In contrast to mammals and yeast, the PKA holoenzyme in *Dictyostelium* consists of only one R and one C subunit [76,77]. The binding of cAMP to the R subunit results in the release of the C, which is now enzymatically active. In *Dictyostelium* the R subunit expression is low in vegetative cells and increases during development, corresponding to increases in cAMP-regulated kinase activity in crude extracts [78].

Cells in which PKA-R is inactivated, or in which PKA-C is constitutively active, develop rapidly, suggesting that PKA regulates the timing of development. Expression of ACA in PKA-C/null cells restores some receptor and GTPγS stimulated activities. On the other hand, the over-expression of the PKA-C in the absence of cAMP pulses is sufficient for the expression of all essential aggregative and postaggregative genes, as demonstrated by the normal development of cells lacking adenylyl cyclase A, but carrying multiple copies of the act15:: PKA-C construct [79]. The most likely explanation is that external cAMP uses two independent signal transduction pathways, one of which requires activation of PKA, while the other is PKA-independent [80,81].

The levels of intracellular cAMP and PKA activity are controlled by the rate of synthesis and degradation of cAMP, the latter controlled by the cAMP-specific phosphodiesterase RegA. Phosphorylation by ERK2 inhibits RegA activity and consequently intracellular cAMP will rise and activate PKA. PKA in turn inhibits ERK2 in an inhibitory loop [82,83] and, directly or indirectly, phosphorylates the receptor cAR1, causing the loss of ligand binding [46]. Cessation of adenylyl cyclase activation and increased RegA activity lower the cAMP level, thus PKA is inhibited, while protein phosphatases return cAR1 to its high-affinity state [83]. The phenotypes of RegA null cells, which have elevated cAMP levels [75] and presumably elevated PKA activity, are similar to those of PKA regulatory subunit (PKA-R) null cells, which have constitutive PKA activity [84,85,86].

Protein degradation plays a central role in controlling PKA activity and cell-type specification in *Dictyostelium*. A model in which the MAP kinase ERK2 controls the degradation of RegA through an SCF complex containing the cullin CulA, and the F-box/WD40-repeat-containing protein FbxA was described [28].

In mammalian, the localization of PKA in the cell is mediated by scaffolding proteins, namely A-kinase anchoring proteins (AKAPs). AKAPs belong to a group of structurally different proteins that share the common feature to target the PKA holoenzyme in close proximity of its substrate [87]. Each AKAP contains a PKA-binding motif that binds the R subunit of PKA and a targeting domain that directs the kinase to specific subcellular compartments. AKAPs form a macromolecular complex, named transduceosome, which assembles components of cAMP generating systems (receptors and ACs), effectors (PKA and Epac) and attenuating enzymes (PDEs and PPs). This implies that complexes nucleated by AKAPs create intracellular domains where distinct signalling pathways converge and are locally attenuated or amplified, optimizing the biological response to extracellular stimuli [88,89].

The Ubiquitin Proteasome System (UPS) can regulate PKA stability and signalling. Praja2 is a widely expressed mammalian atypical RING finger protein with E3 ligase activity. Praja2 acts as an AKAP that binds and targets PKA holoenzyme to the cell membrane, perinuclear region, and cellular organelles. Co-localization of Praja2-PKA complexes with PKA substrate/effector molecules ensures the efficient integration and propagation of the locally generated cAMP to distinct target sites. In course of agonist stimulation, Praja2 couples ubiquitination to proteolysis of the R subunits of PKA. By decreasing the ratio between R/C levels, Praja2 sustains downstream signals carried out by PKA, positively impacting specialized cell functions [90].

AKAP homologs in *Dictyostelium* are missing, and whether PKA regulation is under the control of the ubiquitin-proteasome system is currently unknown.

Optimal gene expression requires periodic, not continuous, cAMP stimulation. The mechanism allowing for distinguishing between periodic and continuous signalling has been recently discovered [91]. A GATA family transcription factor, GtaC, is essential for the regulation of this process. Indeed, GtaC was found to accumulate in the nucleus or disperse in the cytosol in a periodic fashion in response to cAMP waves. The nucleocytoplasmic shuttling requires a coordinated action of a Nuclear Localization Signal (NLS) and the reversible phosphorylation status of the transcription factor, achieved by the GSK3 orthologue, GskA. Interestingly, when this circuit is artificially interrupted by inhibiting GtaC exit from the nucleus, precocious expression of developmentally regulated genes, such as *csA* and *car1*, is induced. The activity of mammalian GATA transcription factors is regulated by phosphorylation, acetylation, but also ubiquitination either in a proteasome-dependent or -independent manner [92,93]. The current findings point-out that *Dictyostelium* GtaC activity and subcellular localization is controlled by phosphorylation, but other mechanisms may be evoked, including ubiquitination itself.

## 6. Is cAR1 Dependent Signalling Pathway Regulated by Ubiquitination?

By making use of random suppression mutagenesis of a *Dictyostelium* aggregation-deficient mutant HSB1, defective in Pia/Rictor dependent ACA activation [94], we recently isolated two suppressor mutants in which the wild type phenotype was restored. Surprisingly, the subsequent analysis of the interrupted genes revealed them to be the same. The gene encodes for a novel giant E3 ubiquitin ligase with an ubiquitin ligase domain homologous to the HECT domain of mammalian HERC1, a SPRY domain—common feature of giant HECT ubiquitin ligases HERC1 and HERC2—but with a PH domain instead of the most typical mammalian RLD domains. Therefore, the protein was named HectPH1 [32]. Although HECT E3 ubiquitin ligases appear to regulate many physiological processes, including membrane receptor and transporter trafficking, mTOR signalling, and transcription or chromatin remodelling, the exact function of HERC1 and HERC2 in mammals remains unclear [13,95,96]. The characterization of the *Dictyostelium* suppressor mutant suggests that HectPH1 might act at different levels of the GPCR signal transduction cascade. The mutant was indeed able to spontaneously chemotax, to relay cAMP, albeit at low level, to restore TORC2-dependent PKB phosphorylation and to express cAMP-dependent aggregation-specific genes. Consistent with Pia/Rictor being defective, adenylyl cyclase ACA was not activated in the suppressor mutant.

Since the most outstanding feature of the suppressor mutant is the hypersensitivity to cAMP, we hypothesized that HectPH1 could regulate one or more elements involved in cAR1 signaling pathways. cAR1 internalization remains still a controversial issue, though there is some evidence that cAR1 undergoes internalization during the aggregation stage [97,98]. Remarkably, when aggregating cells are stimulated with high concentrations of cAMP, the number of cAMP-containing endocytic vesicles increases dramatically [49]. After the aggregation stage, in the aggregated mounds, there is rise in extracellular cAMP, with the high level of cAMP controlling post-aggregative development. Interestingly, the high affinity cAR1 receptors in the mounds are down-regulated and replaced by the low affinity cAR2 and cAR4 receptors. Removal of cAR1 could be controlled by ubiquitination, besides down-regulation at gene level, and cAR1 could be a putative target of HectPH1.

HectPH1 could lead to ubiquitination of cAR1 or of the arrestin AdcC, as it occurs in a number of mammalian GPCRs (e.g., CXCR4), such that its inactivation could result in a higher number of cAR1 receptors being exposed to the cell surface. It will be thus of interest to assess whether cAR1 is directly ubiquitinated, and to what extent ubiquitination might affect the receptor activity and/or its internalization.

In favour of the hypothesis that HectPH1 might regulate cAR1 signaling, rather recently it has been reported in mammalian cells that HERC2 targets the deubiquitinating enzyme USP33, which is involved in regulating β2 adrenergic receptor recycling and re-sensitization [99].

**Figure 4:**
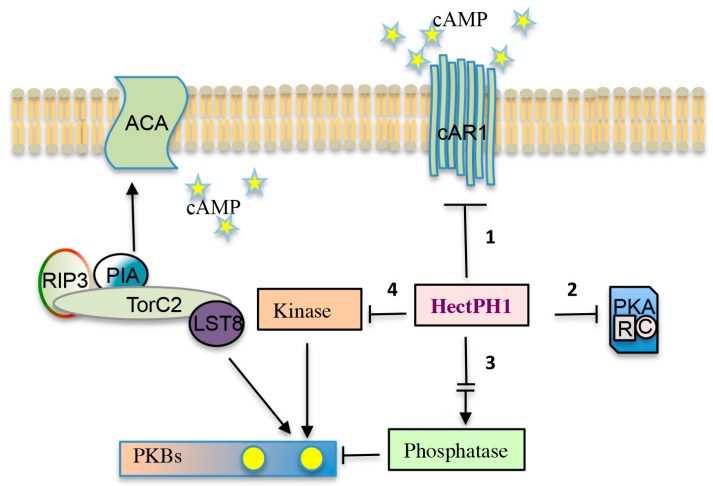
Model of the putative HectPH1 ubiquitin targets in cAMP signalling. Solid arrows and T-bars symbolize positive and negative regulatory loops, respectively. The broken arrow indicates an indirect activation because likely mediated by unknown factor/s.

As mentioned, among the features characterizing the suppressor mutant there was a fully restored PKB activity, suggesting that HectPH1 might be a good candidate as pivotal regulator within the Ras-PI3K-TORC2-PKB pathway. What is its exact role in this scenario is currently unknown but plausible explanations can be envisaged (Figure 4). HectPH1 might regulate a PKB specific phosphatase, such as it occurs in mammalian for PHLPP that is under the control of a RING ubiquitin ligase [68]; otherwise, it can act at the level of an unknown negative regulator of TORC2 or a parallel kinase performing a similar function than TORC2, such as DNA-PK. An alternative option is that HectPH1 regulates the stability of Pia/Rictor, thus leading to an accumulation of the protein resulting in a higher TORC2 kinase activity. This option cannot be excluded, though it seems unlikely, given that Pia/Rictor dependent ACA stimulation by cAMP in the suppressor mutant was not restored.

This notwithstanding, the ACA direct downstream effector, PKA, was activated as well as the expression of aggregation-specific genes. Whether *Dictyostelium* PKA regulation is under the control of the ubiquitin-proteasome system, as it occurs in mammals, is currently unknown and needs to be determined. It is worth mentioning that, although ACA was not activated by cAMP pulsing in the suppressor mutant, the adenylyl cyclase B is however expressed and could be responsible for a basal level of cAMP to be produced in the mutant. Likely, the low cAMP levels are sufficient to stimulate PKA and to activate the cAMP relay, suggesting that HectPH1 inactivation increases the sensitivity to cAMP signals, either at receptor level or in downstream pathway(s) [32].

The targets of the HectPH1 E3 ubiquitin ligase upon cAR1 stimulation are depicted. The HectPH1 could ubiquitinate: (1) directly cAR1, or proteins involved in endocytosis (e.g., arrestins), therefore stimulating receptor de-sensitization and degradation; (2) components of the PKA signalling pathway, transcription factors, such as GtaC, or other components regulating developmental gene expression; (3) a factor activating a phosphatase antagonistic to TORC2, thus regulating PKB phosphorylation; and, (4) a kinase alternative to TORC2.

## 7. Conclusions

Accumulating evidence suggests the ubiquitination and deubiquitination processes are pivotal in regulating different signal transduction pathways, including receptor tyrosine kinases.

More recently, their role in regulating GPCRs in mammals is emerging. The potential outcomes of GPCRs ubiquitination are manifold though the major is the regulation of receptor trafficking to lysosomes. However, more questions regarding the nature of the ubiquitin-dependent sorting machinery, and how such highly dynamic processes are regulated, remain largely unanswered. In addition to its role in regulating receptor trafficking to the lysosomal compartment, and negatively regulating GPCRs internalization, additional functions for ubiquitination in controlling GPCRs trafficking are possible, such as regulating GPCRs membrane transport from the endoplasmic reticulum to the plasma membrane [52]. Furthermore, GPCRs ubiquitination may likely regulate multiple protein-protein interaction thus influencing downstream GPCR activities.

*Dictyostelium* is a preferred model system to investigate GPCR linked pathways, particularly concerning chemotaxis and the control of gene expressionThe genome of the social amoeba encodes a comprehensive repertoire of the ubiquitin-system components very closely related to that of mammals, forecasting the possibility of using *Dictyostelium* as useful model organism to understand the role of ubiquitination in the regulation of the signaling cascades downstream of GPCRs, answering some open questions. In this context, the further analysis of the recently discovered E3 ubiquitin ligase HectPH1, together with the generation of conditional COP9 signalosome mutants and the identification of their potential substrates/interacting partners will be, and may be, very useful in dissecting the role of HERC1-like ubiquitin ligase and COP9 in GPCRs, cAR1, and cAR3, mediated signalling.

## Supplementary Materials

The following are available online at www.mdpi.com/1422-0067/18/10/2180/s1.
