# Supplementary material for: G-Protein Dependent Signal Transduction and Ubiquitination in Dictyostelium
Source: Int J Mol Sci. 2017 Oct 19;18(10):2180. doi: 10.3390/ijms18102180 (PMC5666861; doi:10.3390/ijms18102180)
Supplement: Supplementary file 1 [file ijms-18-02180-s001.pdf]

Table S1. Summary of Dictyostelium Ubiquitin-System members. Different members have been classified into the following families: I) De-ubiquitinating (DUB); II) E1-; III) E2- and IV) E3-ubiquitin ligases -enzymes. IDs refer to Dictybase.org.

	ID
DUB	DDB_G0268872
	DDB_G0269694
	DDB_G0270416
	DDB_G0271264
	DDB_G0271348
	DDB_G0271798
	DDB_G0274207
	DDB_G0274795
	DDB_G0274827
	DDB_G0275021
	DDB_G0275415
	DDB_G0276443
	DDB_G0278929
	DDB_G0282463
	DDB_G0284381
	DDB_G0289611
	DDB_G0290453
	DDB_G0291239
	DDB_G0292046
	DDB_G0293770
	DDB_G0282953
	DDB_G0292290
	DDB_G0293508
	DDB_G0289557
	DDB_G0278795
	DDB_G0282007
	DDB_G0285527
	DDB_G0277251
	DDB_G0285907
	DDB_G0267906
	DDB_G0279375
	DDB_G0271346
	DDB_G0284757
	DDB_G0269646
	DDB_G0272566
	DDB_G0284037
	DDB_G0284597
	DDB_G0277927
	DDB_G0281153
	DDB_G0274229
	DDB_G0293254
	DDB_G0293180
	DDB_G0279633
	DDB_G0267980

E1	DDB_G0268496
	DDB_G0270272
	DDB_G0271096
	DDB_G0277047
	DDB_G0279641
	DDB_G0283891
	DDB_G0286919
	DDB_G0287965
	DDB_G0293306

E2	DDB_G0268938
	DDB_G0271726
	DDB_G0274313
	DDB_G0274897
	DDB_G0275787
	DDB_G0277267
	DDB_G0278775
	DDB_G0280091
	DDB_G0280347
	DDB_G0281369
	DDB_G0281833
	DDB_G0282783
	DDB_G0284009
	DDB_G0284865
	DDB_G0286511
	DDB_G0288345
	DDB_G0288381
	DDB_G0288697
	DDB_G0289021
	DDB_G0290547
	DDB_G0290629
	DDB_G0291199
	DDB_G0292596
	DDB_G0294380
	DDB_G0269724
	DDB_G0286797
	DDB_G0268840
	DDB_G0277319
	DDB_G0289037
	DDB_G0288343

	DDB_G0267718
	DDB_G0267870
	DDB_G0268162
	DDB_G0268860
	DDB_G0268864

	DDB_G0269184
	DDB_G0269486
	DDB_G0269498
	DDB_G0269614
	DDB_G0269996
	DDB_G0270000
	DDB_G0270014
	DDB_G0270200
	DDB_G0270206
	DDB_G0271294
	DDB_G0272082
	DDB_G0272202
	DDB_G0272234
	DDB_G0272602
	DDB_G0273077
	DDB_G0273141
	DDB_G0273399
	DDB_G0273535
	DDB_G0273609
	DDB_G0274241
	DDB_G0274307
	DDB_G0274613
	DDB_G0274743
	DDB_G0274829
	DDB_G0274875
	DDB_G0275005
	DDB_G0276489
	DDB_G0276809
	DDB_G0278133
	DDB_G0278141
	DDB_G0278501
	DDB_G0278617
	DDB_G0278763
	DDB_G0278765
	DDB_G0278979
	DDB_G0278981
	DDB_G0279345
	DDB_G0280089
	DDB_G0280513
	DDB_G0280603
	DDB_G0280707
	DDB_G0280987
	DDB_G0281183
	DDB_G0281595
	DDB_G0281679
	DDB_G0282115

E3	DDB_G0282185
	DDB_G0282479
	DDB_G0282693
	DDB_G0283425
	DDB_G0283971
	DDB_G0284599
	DDB_G0284853
	DDB_G0285149
	DDB_G0285333
	DDB_G0285523
	DDB_G0285581
	DDB_G0285705
	DDB_G0285763
	DDB_G0286803
	DDB_G0286991
	DDB_G0287171
	DDB_G0287629
	DDB_G0287847
	DDB_G0288453
	DDB_G0288525
	DDB_G0288619
	DDB_G0289043
	DDB_G0289719
	DDB_G0290267
	DDB_G0290883
	DDB_G0290931
	DDB_G0290965
	DDB_G0290971
	DDB_G0291011
	DDB_G0291606
	DDB_G0292120
	DDB_G0292312
	DDB_G0292408
	DDB_G0292642
	DDB_G0293132
	DDB_G0290179
	DDB_G0283983
	DDB_G0287221
	DDB_G0276527
	DDB_G0286931
	DDB_G0285063
	DDB_G0287859
	DDB_G0288093
	DDB_G0283475
	DDB_G0267384
	DDB_G0284903

	DDB_G0292794
	DDB_G0291972
	DDB_G0268992
	DDB_G0272775
	DDB_G0285223
	DDB_G0285349
	DDB_G0288223
	DDB_G0292470
	DDB_G0280113
	DDB_G0286909
	DDB_G0276377
	DDB_G0285601
	DDB_G0269230
	DDB_G0273251
	DDB_G0273615
	DDB_G0284889
	DDB_G0276047
	DDB_G0283893
	DDB_G0287859
	DDB_G0288093
	DDB_G0288473
	DDB_G0290179
	DDB_G0292022
	DDB_G0292128
	DDB_G0292264
	DDB_G0268536
	DDB_G0272386
	DDB_G0275493
	DDB_G0275911
	DDB_G0276149
	DDB_G0276887
	DDB_G0280141
	DDB_G0280581
	DDB_G0280955
	DDB_G0281237
	DDB_G0282285
	DDB_G0282667
	DDB_G0285445
	DDB_G0287415
	DDB_G0290985
	DDB_G0292134
	DDB_G0292134
	DDB_G0285601

The GPCR Superfamily

**Family**	**1**	**2**	**3**	**4**	**5**	**6**
(e.g.)	α/β adrenergic Light/Odorant	Glucagon Secretin	GABAb metabotropic glutamate like	Pheromone	Frizzled -Smoothened like	cAR/Crl
**D n. members**	**-**	**1**	**17**	**-**	**25**	**12**
**Organism**	**(M)**	**(M/D)**	**(M/D)**	**(M)**	**(M/D)**	**(M/D)**

FigureS1. Summary and comparison of the Dictyostelium and mammalian GPCR family members: GPCR family members have been classified, and compared, according to their amino acids sequences and structural features. Mammals (M), *Dictyostelium* (D).
